# Effects of Phytase Source and Dose on Its Stability during Pelleting Process under Different Conditioning Temperatures

**DOI:** 10.3390/ani13233741

**Published:** 2023-12-03

**Authors:** Yuming Wang, Feng Zhao, Hu Zhang, Qianyun Zhang, Wei Zhao, Renna Sa, Jingjing Xie

**Affiliations:** 1 State Key Laboratory of Animal Nutrition and Feeding, Institute of Animal Sciences, Chinese Academy of Agricultural Sciences, Beijing 100193, China; wangyuming@caas.cn (Y.W.); zhaofeng@caas.cn (F.Z.); ndzhanghu@163.com (H.Z.); sarenna@caas.cn (R.S.); 2 Provincial Key Agricultural Enterprise Research Institute of Encapsulated Feed Additive, King Techina Technology Co., Ltd., Hangzhou 311107, China; zhangqianyun_kdq@126.com (Q.Z.); zhaowei_kdq@126.com (W.Z.)

**Keywords:** conditioning temperature, dose, phytase, recovery rate of activity, source

## Abstract

**Simple Summary:**

Phytase activity is affected by extreme pressure and temperature during feed processing, resulting in variable residual activity and efficacy on the digestibility of phosphorus when applied to pelleted feed. The results of this study indicate that phytase from different sources has variable thermal stability when subjected to pelleting, but the dose does not influence the recovery rate of phytase. Furthermore, our findings suggest that the recovery rate of phytase decreased when the conditioning temperature increased from 75 to 85 °C. These findings may inform decision-making processes when applying phytase and pelleting to feed.

**Abstract:**

Phytase activity can be impaired during pelleting because of extreme thermal conditions. This study investigated the effects of dose and source of phytase on phytase activity during the conditioning, pelleting, and cooling process. A split-plot design was used in two experiments, with five phytase doses (Exp. 1; 7560, 14310, 33830, 43590 and 61500 FTU/kg) or eight phytase sources (Exp. 2) as the main plot and steam conditioning temperatures (Exp. 1 and 2; 75 and 85 °C) as the subplot. Each treatment processed four batches, one batch per replicate. The results of Exp. 1 showed phytase dose in diets had no effect (*p* > 0.05) on the recovery rate of phytase activity after the conditioning, pelleting, or cooling process. The recovery rate of phytase activity in each process was higher (*p* < 0.05) at 75 °C than that at 85 °C for both Exp. 1 and 2. The phytase source significantly affected (*p* < 0.05) the recovery rate of phytase activity and had varied appearances of structure. In conclusion, the structure, phytase activity, and phytase recovery after steam conditioning–pelleting significantly varied across sources, but the stability of phytase was not affected by dose.

## 1. Introduction

Phytate (Inositol hexakisphosphate) is present in most cereal grains in the form of complexes with minerals and amino acids, and it impairs the bioavailability and nutritional value of the feed. Phytase can degrade phytate, increase the digestibility of phosphorus, minerals, and amino acids, and reduce excretion of inorganic phosphorus [1,2,3]. At present, a powdered phytase is treated as a regular ingredient, which is mixed with the main ingredients before the steam conditioning–pelleting process [3]. Typically, the feed mixture is steam conditioned to gelatinize starch and sterilize feed, before being processed into pellets [4]. The stability of phytase during the pelleting process is a major concern because phytase activity is negatively affected by pressure and high temperature [5]. Phytase must be biologically active when reaching the gastrointestinal tract to be able to act on the substrates. Therefore, understanding the loss of phytase activity during the steam conditioning–pelleting process and the variability of stability across sources is important to assess in order to ensure adequate digestible phosphorus in animal feeds [6,7].

Previous studies evaluating phytase stability assumed that the initial dietary phytase concentration in the unprocessed diet did not affect the recovery rate of phytase activity after processing, resulting in a wide variation in phytase activity (varied from 376 to 20,220 FTU/kg) [4,8,9,10]. However, the study of Boney and Moritz [11] revealed that the residual activity of phytase added to broiler diets at 6-fold the standard dose was about 6-fold the residual activity of phytase at the standard dose after steam conditioning–pelleting, but 4-fold greater than some other treatments. Another study showed that the recovery rate of phytase activity after 3 min in a water bath at 80 ℃ decreased when the initial activity of phytase decreased, which implied that phytase stability depends on the phytase dose [8]. These variable results warrant further investigations to clearly elucidate the effect of initial phytase dose on stability. Additionally, others report substantial differences in the stability of phytase across different sources and different conditions used in pelleting [12], further complicating interpretations of the previous findings.

Currently, few published studies evaluated the stability of commercially available phytase sources exposed to various temperatures during pelleting. It is hypothesized that differences in phytase doses may contribute to the variation in observed enzyme stability. Hence, this study systematically evaluated the effects of phytase dose on the recovery rate of phytase activity, to determine a suitable dose range to assess heat stability of phytase in corn–soybean meal diets. The thermal stability of eight phytases commercially available in China was then evaluated using steam conditioning–pelleting based on the above results.

## 2. Materials and Methods

### 2.1. Phytase Source

The phytase used in Exp. 1 was 6-phytase, which is derived from a liquid fermentation product of the phytase gene from *Escherichia coli* and expressed in *Pichia pastoris*. The enzyme was granularized via spray drying, and the labeled phytase activity was 10,000 FTU/g. In Exp. 2, 8 phytase products declared potency of 10,000 FTU/g and were collected from Chinese feed manufacturers: Premier Ⅰ (SunSon Industry Group Co., Ltd., Ningxia, China); Sunfize 10,000 (Wuhan Sunhy Biology Co., Ltd., Hubei, China); Microtech 10,000 Plus (Guangdong VTR Biotech Co., Ltd., Guangdong, China); Pecozyme Phytase (Challenge Group, Beijing, China); Winovazyme Phytase HT85 (Beijing Winovazyme Biotech Co., Ltd., Beijing, China); Kingphos (Qingdao Vland Biotech INC., Shandong, China); Natuphos (BASF Corporation, Florham Park, NJ, USA); Axtra Phy (Danisco Animal Nutrition, Marlborough, UK). These phytases were randomly numbered 1 to 8 for reference and included 6 powder samples and 2 pellet spheres.

### 2.2. Experimental Design

This study consisted of 2 experiments.

#### 2.2.1. Exp. 1

A split-plot 5 × 2 factorial design was used to determine the effects of phytase doses and steam conditioning temperatures on the stability of phytase. Five dietary phytase doses (7560, 14,310, 33,830, 43,590, and 61,500 FTU/kg) were the main plot, and 2 conditioning temperatures (75 and 85 °C) were the subplot. To prevent residues of the higher phytase doses in the pelleting machine contaminating the lower dose pellets, repeated sample batches were produced each day for 5 days, from least to greatest dose. Four batches were produced daily at 08:00–9:30, 10:00–11:30, 13:00–14:30, and 15:00–16:30, resulting in four replicates per treatment for each dose.

#### 2.2.2. Exp. 2

A split-plot 8 × 2 factorial design was used to determine the effects of phytase sources and steam conditioning temperatures on phytase stability. Eight phytase sources were the main plot and 2 conditioning temperatures (75 and 85 °C) were the subplot. A basal diet without phytase was used resulting in a 4 × 9 Youden square design with 4 batches of 9 diets (a basal diet and 8 experimental diets). The 8 experimental diets were formulated by adding 0.4% phytase from 8 sources in the basal diet. Taken production batches were blocked to give 4 replicates per diet (Table 1).

### 2.3. Experimental Diet

Experimental diets were composed of corn and soybean meal, limestone, dicalcium phosphate, phytase, sodium chloride, crystalline amino acids, and a vitamin and mineral premix (Table 2). Corn and soybean meal were ground and screened through a 2 mm sieve; the remaining ingredients were then mixed with ground corn and soybean meal step by step in equal proportion, and then put into the double-circle paddle mixer (FAMSUN SJHSO.2, Jiangsu Muyang Group Co., Ltd., Yangzhou, China), and mixed diets were pelleted through a pellet mill as described below.

### 2.4. Steam Conditioning–Pelleting Process and Sample Collection

The steam conditioning of homogenized diets was carried out using a double-shaft steam conditioner (FAMSUN SBTZ10, Jiangsu Muyang Group Co., Ltd., Yangzhou, China; conditioner cylinder = 1.2 m × 22 cm). The mixture was conditioned for 70 s at a constant temperature of approximately 75 or 85 °C. The length-to-diameter ratio of the pellet mill (FAMSUN SZLH180X25, Jiangsu Muyang Group Co., Ltd., China) was 6:1 with a 4 mm diameter hole and a production rate up to 100 kg/h. Two electronic temperature sensors located at the conditioner and the pellet mill discharge port logged actual temperature continuously. After pelleting, diets were cooled to room temperature using a pilot-scale forced air dryer.

The mixer, storage bin, and conditioner were thoroughly cleaned with high-pressure air to remove residual feed after each conditioning–pelleting session. When the conditioning temperature reached the target temperature (75 or 85 °C), the pellet mill operated constantly for 15 min. Samples were taken continuously for 10 min at the feeder sampling port before conditioning, after conditioning, after pelleting, and after cooling. One sample was collected every minute, and 10 subsamples were collected at each sampling site.

The steam conditioning–pelleting process was performed as follows: the main steam valve and compressed air valve were opened, diets were mixed in the mixer for 5 s, diets were transferred to the pelleting buffer bin, and the steam valve and drain valve sequentially opened and then closed after discharging cold water. The pellet mill, conditioner, and pellet feeder were computer controlled. The samples collected after each process were immediately refrigerated (4 °C) until analysis.

### 2.5. Determination of Feed and Phytases

The particle shape, color, and appearance of the phytase were observed using a stereomicroscope (Leica M205 FA, Wetzlar, Germany). The temperatures during the conditioning and pelleting process were automatically recorded using sensors at the discharging port of the conditioner and pelleting mill. Dry matter content of the feed samples was determined according to AOAC method 930.15. The phytase activity of concentrated phytase supplements and diets was determined using the colorimetric measurement of the released phosphate [13,14]. In both cases, the 1 FTU of phytase was defined as 1 μmol of inorganic phosphorus released from 5.0 mmol/L sodium phytate per minute at pH 5.50 and 37 °C.

### 2.6. Data Calculation and Statistical Analysis

All data were assessed with SAS 9.4 (SAS Inst. Inc., Cary, NC, USA). Differences among treatments in the measured conditioning temperature and dry matter content of diets were analyzed using the GLM model and Tukey’s multiple range test.

The recovery rate of phytase activity was defined as the ratio of the dietary phytase activity assessed after each processing phase to that assessed in raw (unprocessed) feed. According to the completely randomized split-plot design principle described by Kaps and Lamberson [15], PROC MIXED was used to analyze the differences in the recovery rate of phytase activity, with processing batch as the experimental unit. The statistical model of Exp. 1 assessed the dose of phytase, conditioning temperature, and their interaction as the main effect, and the random effect of block. The statistical model of Exp. 2 included the source of phytase, conditioning temperature, and their interaction as the main effects, and the random effect of block. Differences among treatments were compared using Tukey’s test. Only the main effects were discussed for responses in which the interaction was not significant, whereas the whole treatments were discussed for responses where the interaction was detected. For all analyses, *p* < 0.05 was considered significant.

## 3. Results

### 3.1. Effect of Phytase Dose on Recovery Rate of Phytase Activity (Exp. 1)

The actual steam conditioning temperature ranged from 75.1 to 75.3 °C or 84.6 to 85.0 °C when set at 75 or 85 °C, respectively, and there was no difference among treatments in Exp. 1 (Table 3). No difference was detected across treatments for dry matter content of the diets after conditioning, pelleting, or cooling when conditioned at 75 °C. However, the dry matter in the phytase dose of 61,500 FTU/kg was greater (*p* < 0.05) than that of 14,310 FTU/kg after the conditioning process when conditioned at 85 °C, but no other differences (*p* > 0.05) were detected among the treatments when conditioned at 85 °C. While statistically significant, this difference related to the small coefficient of variation within replicates and the SEM among treatments and is not practically significant.

**Figure 1:**
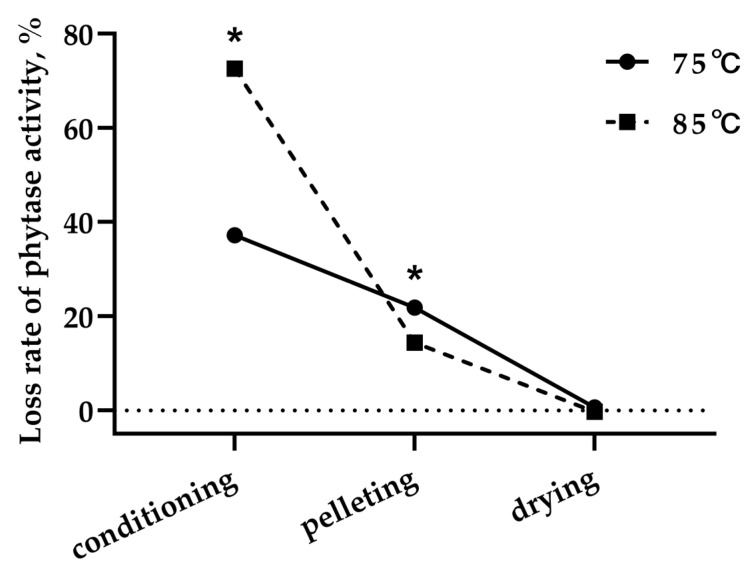
Loss rate of phytase activity during the steam conditioning–pelleting and cooling process. * Significant differences between treatments.

No dose of interactive effect (*p* > 0.05) between phytase activity and conditioning temperature was found for the recovery rate of phytase activity after conditioning, pelleting, or cooling (Table 4). The dose of phytase in diets had no effect (*p* > 0.05) on the recovery rate of phytase activity after conditioning, pelleting, or cooling, whether conditioned at 75 or 85 °C. The recovery rate of phytase activity after conditioning, pelleting, and cooling was greater (*p* < 0.05) for diets conditioned at 75 vs. 85 °C. Consequently, the loss of phytase activity during the conditioning step was substantially greater at 85 °C than 75 °C (*p* < 0.05). However, the opposite result was observed during the pelleting step (Figure 1), but the difference is comparatively small in magnitude compared to the conditioning step.

### 3.2. Characteristics of Phytase from Different Sources (Exp. 2)

**Figure 2:**
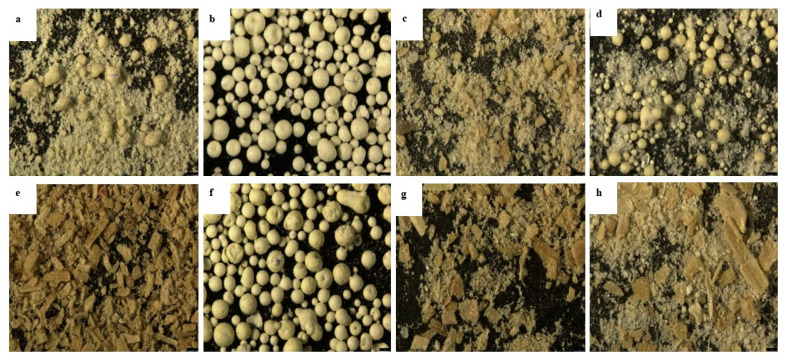
Microscopic photos of phytase products (300×, Exp. 2). These photos of the phytase products were taken using a stereomicroscope (Leica M205 FA, Wetzlar, Germany) at a 300× of view. (**a**) Phytase 1; (**b**) Phytase 2; (**c**) Phytase 3; (**d**) Phytase 4; (**e**) Phytase 5; (**f**) Phytase 6; (**g**) Phytase 7; (**h**) Phytase 8.

The eight phytase samples from different manufacturers differed in appearance under the microscope: two samples were spherical particles (Figure 2b,f) and six were powders (Figure 2). Among the six powder samples: one contained many spherical particles (Figure 2d), three contained a few spherical particles (Figure 2a,c,h), and two appeared as uniformly fine powder (Figure 2e,g). All eight phytases were yellow or pale yellow.

The determined phytase activity showed that four of the phytases had measured activities greater than 12,000 FTU/g and two were less than 10,000 FTU/g (Table 5). The relative deviation between measured and calculated phytase activity was 7.02% or 5.11% in two sources, but other sources had comparatively less relative deviation. Dietary phytase activity was not different between phytases 2 and 3, but it was significantly higher (*p* < 0.05) than that of other phytases. Measured phytase activities between diets with phytases 1, 4 and 6 were not different (*p* > 0.05), but they were significantly greater (*p* < 0.05) than that with phytase 5. The measured phytase activity of diets with phytase 7 was significantly higher (*p* < 0.05) than that with phytase 5. The measured phytase activities were not different (*p* > 0.05) between diets added with phytases 5 and 8, but they were significantly higher (*p* < 0.05) than diets without phytase. A total of 4.2 FTU/g of activity was measured in the control diet without the addition of phytase.

### 3.3. Effect of Phytase Source on Recovery Rate of Phytase Activity (Exp. 2)

The measured conditioning temperatures of the eight diets with added phytase varied from 74.2 to 75.8 °C when set at 75 °C, and there was no difference (*p* > 0.05) among treatments (Table 6). The measured conditioning temperatures of the eight diets with added phytase varied from 85.0 to 85.4 °C when set at 85 °C, and there was also no difference (*p* > 0.05) among treatments.

No interactive effects (*p* > 0.05) were found between the phytase source and conditioning temperature on the recovery rate of phytase activity after conditioning, pelleting, or cooling (Table 6). The phytase source and conditioning temperature significantly affected (*p* < 0.05) the recovery rate of phytase activity in all processes. After the diets were steam conditioned, the recovery rate of phytase 2, 4, and 5 activities were higher (*p* < 0.05) than that of phytase 6 and 7, but there was no significant difference between the recovery rates of phytase 1, 3, and 8. After steam conditioning and pelleting, the recovery rate of phytase 5 activity was higher (*p* < 0.05) than that of phytase 2, 6, and 7, while there was no difference between the recovery rate of phytase 1, 3, 4 and 8. Subsequently, the pelleted diets were dried. The recovery rate of phytase 5 activity was higher (*p* < 0.05) than that of phytase 2, 6, and 7; but the recovery rate of phytase 1, 2, 3, 4, 6 and 7 did not significantly differ (*p* > 0.05). The recovery rate of phytase activity after conditioning, pelleting and cooling was greater (*p* < 0.05) for diets conditioned at 75 vs. 85 °C.

## 4. Discussion

### 4.1. Differences in Thermal Stability of Phytase at Different Doses

Phytase was recommended at the levels of 500 to 1000 FTU/kg in diets for pigs and poultry when it was introduced as a feed additive [16,17,18]. However, pig trials demonstrated the optimal phytase dose depended on diet composition [19]. For example, the optimal dose of phytase for corn, rapeseed meal, and soybean meal is 500 to 1000 FTU/kg, but that for sunflower meal and wheat is 2000 FTU/kg [19]. Generally, phytase doses greater than 1500 FTU/kg have been considered as supra dosage [20,21,22,23], but a dose of 1000 to 1500 FTU/kg is the current norm [24]. This shift is attributable to the reduced cost of phytase, increased cost of rock phosphate, and increasing use of the steam conditioning–pelleting technique. Previous studies showed that the addition of a super dosage (2000 to 4000 FTU/kg) in diets before feed pelleting improved growth performance, apparently due to the high loss of phytase activity during the steam conditioning–pelleting process. Over supplementing phytase ensures that the residual phytase activity in the pellets is sufficient to ensure complete phytate hydrolysis [22]. Nutritionists need solid data to adjust dietary phytase to account for the degradation of the enzyme during processing. If the percentage of residual phytase activity in pellets is similar across doses, a constant value could be used to adjust the inclusion of phytase in the feed to achieve a desired rate of phytase activity after processing. Walk et al. [20] reported the recovery rates of 92.8%, 97.8%, and 78.4% when adding 500, 1000, or 1500 FTU/kg phytase in broiler diets, respectively. However, the experimental results of Boney and Moritz [11] showed rates of phytase recovery after pelleting were similar when phytase was supplemented at either the standard or 6-fold the standard dose.

In Exp. 1, the required phytase dose was above 7500 FTU/kg, considering the loss of phytase activity and the detection limit calculated by the method stated in the determination of the phytase activity method [14]. Secondly, the reproducibility of phytase activity determination was unacceptable, and the relative standard deviations of repeatability and reproducibility were 2.2% to 10.6% and 5.4% to 15%, respectively [25]. For these reasons, the difference in recovery rate of phytase activity between different replicates of the same treatment was unacceptably high (the absolute different between two replicates can be up to 20%) [26]. Therefore, a high dose of phytase activity reduced the measurement error. This resulted in supra dosage of phytase added in diets (e.g., 10,000 FTU/kg) during the determination of residual phytase activity after steam conditioning and pelleting [27].

Timmons et al. [28] showed that the recovery rate was 64% to 80.0% and 69.5% to 81.0%, when two thermostable phytases were included at 0.5, 1 and 2 times the recommended dose and pelleted with a conditioning temperature of 93 °C. The statistical test showed that the dose had no effect on the recovery rate of phytase activity. In this experiment, the recovery rate of phytase activity varied numerically, but not significantly with doses after conditioning, pelleting, or cooling. This was similar to the results of Timmons et al. [28] and De Jong et al. [4], indicating that the phytase dose in diets did not affect the recovery rate of phytase activity.

### 4.2. Differences in Thermal Stability of Phytase from Different Sources

Genes for commercial phytases are mainly expressed in fungi, such as *Aspergillus niger*, and bacteria, such as *E. coli*, attributing to the variation in molecular weight, optimum pH, and optimum temperature of phytase from different sources [29]. The thermal stability of phytase can be improved via genetic modification, for example, by glycosylation [30]. Therefore, phytase from different genetic sources may differ significantly in terms of heat resistance. In addition, phytase is generally produced in a powdered or granulated form via spray drying and combining with an inert carrier, such as bran, or corn cob powder; but carriers are not uniform [29,31]. The problem with incorporating phytase into animal feed is the denaturation of the enzyme protein caused by moisture and high temperature during the steam conditioning–pelleting process [32]. Some progress has been made towards stabilizing phytase by screening potential encapsulation materials to minimize contact between the enzyme and water, improving its heat resistance during the conditioning process under low-moisture conditions [33,34]. Encapsulation further increases the diversity of phytase properties, in addition to variation from different phytase gene sources, as discussed above [31,35].

In Exp. 2, the phytase from eight different manufacturers showed apparent differences in physical characteristics likely from variation in the processing techniques. There were two spherical particle samples, possibly from spray drying. Four products consisted of a mixture of powder and particles (granulated phytase mixed with a carrier), and there were two fully granulated phytase products. However, it is not clear whether the pelleted phytases were coated with starch or treated with water-repellant coating materials to improve heat resistance. De Jong et al. [10] studied the phytase stability of the commercial phytases from four manufacturers (two powders and two coated phytase) and it varied greatly with storage time under different temperature conditions. The phytase activity loss rate of the coated product was similar to that of the powder samples. This result indicates that the coating did not improve the stability of the enzyme at an ambient temperature. In this experiment, the recovery rate of eight commercial phytase activities after steam conditioning–pelleting clearly stratified into high, medium, and low stability groups. The powdered product 5 had the highest recovery, but granulated product 2 was high in activity after steam conditioning and lost much of its activity after pelleting. The residual activity of particulate product 6 after conditioning–pelleting was the lowest. These results indicated that although pelleting or encapsulation protected phytase from the high temperature and humidity during conditioning, the high pressure, temperature, and shear force during extrusion appeared to disrupt the pellet structure and cause extensive enzyme denaturation. In summary, the encapsulation or pelleting treatments of phytase products from some manufacturers did not significantly improve the heat resistance of the enzymes.

### 4.3. Loss of Phytase Activity during Conditioning, Pelleting, and Cooling Process

The recovery rate of phytase activity after steam conditioning and pelleting in different studies varied greatly from 22.3% to 96.0% [9,28,34]. Firstly, there are differences in the conditions of conditioning and pelleting, such as the conditioning time (range = 10 to 90 s), steam pressure, type of conditioner, and pellet mill die specifications [33]. These conditions affected parameters related to activity loss, such as the conditioning time under high temperature and the moisture change during the pelleting process. Secondly, there are differences in the source of phytase products. Different genes of phytase and post-treatment processes further increase the variation in products from different manufacturers [9,10]. The above studies clearly indicate that phytase has a high sensitivity to steam conditioning and pelleting. In this experiment, when phytases were conditioned at 75 °C and 85 °C, the recovery rates of phytase activity after conditioning were 54.1% to 71.7% and 17.3% to 68.7%, respectively. Increasing the conditioning temperature significantly decreased the recovery rate of phytase activity, and the difference was independent of phytase dose and source. The difference in recovery rate of activity with temperature was caused by greater heat denaturation of phytase at the higher temperature. Most enzyme proteins will be denatured at 60 to 70 °C [32]. The interaction between water molecules and enzyme proteins through non-covalent van der Waals bonds affects enzyme stability during the conditioning–pelleting process [36,37]. Interestingly, the phytases with the highest or the lowest recovery rate of activity after conditioning at 75 °C had the same high or low activity after conditioning at 85 °C. De Jong et al. [4] reported that the recovery rates of four phytase activities after steam conditioning at 75 °C and 85 °C were 21.4% to 78.2% and 3.5% to 43.3%, respectively, and also showed the phytases with a high or low recovery rate of activity after conditioning at 75 °C remained high or low after conditioning at 85 °C. The above results demonstrated substantial differences in heat stability across phytase sources which is exacerbated by elevated temperature.

In Exp. 2, the average recovery of phytase activity was 62.8% and 49.7%, after steam conditioning at 75 °C and 85 °C, respectively, and 36.3% and 26.4% after pelleting. The loss of phytase activity during pelleting was equivalent to 72.6% and 46.1% of the loss during conditioning at 75 °C and 85 °C, respectively, while the loss of phytase activity during cooling was minor (less than 5%). This effect relates the diet temperature rising 3 to 15 °C after pelleting with a greater temperature difference when conditioning at a lower temperature [33,34,37]. During cooling, the hot particle samples were quickly transferred to an air-forced drier for cooling, and the temperature of the samples could be reduced to room temperature within 5 min, and the moisture content was less than 14%. The simultaneous reduction in temperature and moisture in this process is beneficial to the preservation of phytase activity [10], and the cooling process does not significantly impair the phytase activity. Consequently, future work should focus on stability during conditioning and pelleting, with less emphasis on cooling.

## 5. Conclusions

The phytase dose added to animal diets did not affect the recovery rate of phytase activity in either conditioning–pelleting processes. This means that the dose of a particular phytase required to achieve a desired recovery rate of activity after conditioning–pelleting can be determined with reasonable accuracy using a single dose. However, the recovery rate of eight commercial phytase activities varied greatly after the conditioning–pelleting process, so it is critical for nutritionists to know specific expectations for expected recovery. The recovery rate of phytase activity after conditioning and pelleting at 75 °C was higher than that at 85 °C, because of the decreased enzyme inactivation at the lower temperature.

## Figures and Tables

**Table 1:** Batch of production and sequence of steam conditioning–pelleting (Exp. 2).

Batch of Production	No. of Diets								
1	2	4	6	3	7	5	8	1	9
2	7	9	3	2	1	4	6	8	5
3	4	1	7	5	9	3	2	6	8
4	3	5	9	4	8	1	7	2	6

**Table 2:** Ingredient and calculated nutrient composition of experimental diet (as-fed basis, %).

Item	Diet
Ingredients	
Corn	73.71
Soybean meal	23.23
Limestone	0.79
Dicalcium phosphate	1.18
Sodium chloride	0.39
L-lysine∙HCl	0.31
L-threonine	0.09
DL-methionine	0.08
L-tryptophan	0.02
Premix ^1^	0.20
Total	100.00
Nutrient composition ^2^, %	
Dry matter	88.41
Crude protein	16.01
Ether extract	3.89
Crude fiber	2.76
Calcium	0.73
Total phosphorus	0.61
Lysine	1.15
Methionine	0.34
Threonine	0.70
Tryptophan	0.20

^1^ The premix provided the following per kg of diets: VA 1 500 IU, VD_3_ 170 IU, VE 11 IU, VK_3_ 0.5 mg, VB_1_ 1.0 mg, VB_2_ 2.5 mg, VB_6_ 1.0 mg, VB_12_ 11 μg, pantothenic acid 8.0 mg, nicotinic acid 10.0 mg, folic acid 0.3 mg, biotin 0.05 mg, choline chloride 350 mg, Cu (as copper sulfate) 4.5 mg, Fe (as ferrous sulfate) 70 mg, Zn (as zinc sulfate) 70 mg, Mn (as manganese sulfate) 3.0 mg, Se (as sodium selenite) 0.3 mg, I (as calcium iodate) 0.14 mg. ^2^ The chemical compositions were calculated values according to NRC (2012).

**Table 3:** The determined conditioning temperature and dietary dry matter content in steam conditioning–pelleting and cooling process for diets added different doses of phytase (Exp. 1).

Phytase Doses, FTU/kg	Conditioning at 75 °C				Conditioning at 85 °C			
	Temperature, °C	Dry Matter, %			Temperature, °C	Dry Matter, %		
		Conditioning	Pelleting	Cooling		Conditioning	Pelleting	Cooling
7560	75.2	83.58	83.89	85.44	85.0	83.11 ^ab^	83.49	85.10
14,310	75.1	83.63	84.03	85.67	85.0	82.59 ^b^	83.35	85.17
33,830	75.2	83.72	83.82	85.60	84.6	83.00 ^ab^	83.72	85.66
43,590	75.2	84.46	84.73	86.10	85.0	83.89 ^ab^	84.43	86.01
61,500	75.3	84.41	84.89	86.28	85.0	84.15 ^a^	84.43	86.07
SEM	0.07	0.003	0.003	0.003	0.19	0.004	0.003	0.004
*p* value	0.5887	0.1176	0.0923	0.3913	0.6248	0.0349	0.1127	0.2313

Values with the same or no letter superscripts in the same column mean no significant difference (*p* > 0.05).

**Table 4:** Effect of dietary phytase dose and temperature on the recovery rate of phytase activity after steam conditioning–pelleting and cooling process (Exp. 1).

Phytase Dose, FTU/kg	Temperature, °C	Recovery Rate of Phytase Activity, %		
		Conditioning	Pelleting	Cooling
7560	75	60.4	42.6	39.8
	85	39.1	17.4	13.9
14,310	75	65.7	44.2	44.0
	85	36.1	17.0	17.9
33,830	75	63.8	37.0	35.3
	85	26.5	9.2	10.0
43,590	75	64.4	41.6	42.5
	85	18.0	7.4	8.5
61,500	75	60.0	39.7	40.7
	85	17.3	13.9	16.3
SEM		8.0	3.6	3.3
Source of variance				
Phytase dose, *p* value		0.5139	0.3141	0.2384
7560		49.7	30.0	26.9
14,310		50.9	30.6	30.9
33,830		45.2	23.1	22.6
43,590		41.2	24.5	25.5
61,500		38.6	26.8	28.4
SEM		5.7	2.9	2.5
Temperature, *p* value		<0.0001	<0.0001	<0.0001
75 °C		62.8	41.0	40.4
85 °C		27.4	13.0	13.3
SEM		3.6	1.6	1.5
Phytase dose × Temperature, *p* value		0.5352	0.6119	0.5232

**Table 5:** Activity and appearance of phytase products and determined or calculated phytase activity in diets (Exp. 2).

Phytase	Property of Phytase			Phytase Activity in Diet, FTU/g DM		
	Phytase Activity ^1^, FTU/g	Appearance	Color	Determined ^1^	Calculated ^2^	RE ^3^, %
1	11,809	Powder, with a few particle	Pale yellow	56.5 ^bc^	54.5	1.80
2	14,864	Irregular balls, uneven size	Pale yellow	70.8 ^a^	68.4	1.72
3	15,225	Powder, with a few crystalline particle	Pale yellow	73.0 ^a^	70.0	2.10
4	12,094	Powder, with many spherical particles of uneven size	Pale yellow	61.7 ^b^	55.7	5.11
5	9787	Powder, flakes	Yellow	43.2 ^d^	45.1	2.15
6	12,827	Sphere particles, uneven size	Pale yellow	62.4 ^b^	59.1	2.72
7	11,673	Powder, flakes	Yellow	54.1 ^c^	53.8	0.28
8	8058	Powder, flakes	Yellow	42.7 ^d^	37.1	7.02
Without phytase	-	-	-	4.2 ^e^	-	-
SEM				2.0		
Source of variance, *p* value						
Block				0.0141		
Phytase				<0.0001		

Values with the same or no letter superscripts in the same column mean no significant difference (*p* > 0.05). ^1^ The phytase activity for purified phytase and “in feed” phytase was determined using the colorimetric measurement of the released phosphate [13,14]. ^2^ Calculated values of phytase activity in diet = 0.004 g × activity per gram of phytase/DM of diet. ^3^ RE is the relative deviation of the absolute difference between the determined value and the calculated value from the above average value.

**Table 6:** Recovery rate of phytase activity after steam conditioning–pelleting and cooling process (Exp. 2).

Phytase	Temperature, °C	Measured Temperature, °C	Recovery Rate of Phytase Activity, %		
			Conditioning	Pelleting	Cooling
1	75	74.2	61.8	35.5	39.4
	85	85.0	54.7	28.8	28.8
2	75	75.3	64.9	29.0	32.6
	85	85.1	65.6	28.9	26.7
3	75	75.6	59.6	34.7	39.2
	85	85.3	43.0	25.8	26.1
4	75	75.4	71.7	39.9	41.3
	85	85.4	68.7	26.7	27.0
5	75	75.8	71.7	47.2	54.4
	85	85.3	63.8	36.6	36.8
6	75	75.2	54.1	35.9	39.7
	85	85.0	33.1	21.4	20.6
7	75	74.7	55.5	31.4	33.0
	85	85.1	26.3	14.7	12.7
8	75	75.6	64.8	36.9	43.5
	85	85.3	42.8	28.7	26.6
SEM		1.3	6.5	3.7	3.7
Source of variance					
Phytase, *p* value			0.0001	0.0028	<0.0001
1			58.3 ^ab^	32.1 ^ab^	34.1 ^abc^
2			65.3 ^a^	29.0 ^b^	29.6 ^bc^
3			51.3 ^ab^	30.3 ^ab^	32.6 ^bc^
4			70.2 ^a^	33.3 ^ab^	34.2 ^abc^
5			67.7 ^a^	41.9 ^a^	45.6 ^a^
6			43.6 ^b^	28.6 ^b^	30.2 ^bc^
7			40.9 ^b^	23.1 ^b^	22.8 ^c^
8			53.8 ^ab^	32.8 ^ab^	35.0 ^ab^
SEM			5.1	2.7	2.9
Temperature, *p* value			<0.0001	<0.0001	<0.0001
75 °C			63.0	36.3	40.4
85 °C			49.7	26.4	25.7
SEM			3.8	1.7	2.2
Phytase × Temperature, *p* value			0.1386	0.4017	0.3385

Values with the same or no letter superscripts in the same column mean no significant difference (*p* > 0.05).

## Data Availability

The data presented in this study are available on request from the corresponding author.
